# Marine-derived microbes and molecules for drug discovery

**DOI:** 10.1186/s41232-022-00207-9

**Published:** 2022-06-03

**Authors:** Yoshimasa Tanaka, Megumi Nishikawa, Kaho Kamisaki, Saki Hachiya, Moeka Nakamura, Takahiro Kuwazuru, Susumu Tanimura, Kiyoshi Soyano, Kohsuke Takeda

**Affiliations:** 1 Center for Medical Innovation, Nagasaki University, 1-7-1 Sakamoto, Nagasaki, 852-8588 Japan; 2 Department of Cell Regulation, Graduate School of Biomedical Sciences, Nagasaki University, 1-14 Bunkyo-machi, Nagasaki, 852-8521 Japan; 3 Institute for East China Sea Research, Nagasaki University, Bunkyo-machi, Nagasaki, 852-8131 Japan

**Keywords:** Autoimmune disease, Autoinflammatory disease, Biologic, Conventional drug, Marine microbe, Monoclonal antibody, Nanobody, Rheumatoid arthritis, Shark new antigen receptor

## Abstract

Increasing attention has been paid to marine-derived biomolecules as sources of therapeutics for autoimmune diseases. Nagasaki Prefecture has many islands and is surrounded by seas, straits, gulfs, bays, and coves, giving it the second longest coastline in Japan after Hokkaido. We have collected more than 20,000 marine microbes and have been preparing an original marine microbial extract library, which contains small and mid-size biomolecules that may penetrate cell membranes and interfere with the intracellular protein–protein interaction involved in the development of autoinflammatory diseases such as familial Mediterranean fever. In addition, we have been developing an indoor shark farming system to prepare shark nanobodies that could be developed as potential therapeutic agents for autoimmune diseases. Sharks produce heavy-chain antibodies, called immunoglobulin new antigen receptors (IgNARs), consisting of one variable domain (V_NAR_) and five constant domains (C_NAR_); of these, V_NAR_ can recognize a variety of foreign antigens. A V_NAR_ single domain fragment, called a nanobody, can be expressed in *Escherichia coli* and has the properties of an ideal therapeutic candidate for autoimmune diseases. Shark nanobodies contain complementarity-determining regions that are formed through the somatic rearrangement of variable, diversity, and joining segments, with the segment end trimming and the N- and P-additions, as found in the variable domains of mammalian antibodies. The affinity and diversity of shark nanobodies are thus expected to be comparable to those of mammalian antibodies. In addition, shark nanobodies are physically robust and can be prepared inexpensively; as such, they may lead to the development of highly specific, stable, effective, and inexpensive biotherapeutics in the future. In this review, we first summarize the history of the development of conventional small molecule drugs and monoclonal antibody therapeutics for autoimmune diseases, and then introduce our drug discovery system at Nagasaki University, including the preparation of an original marine microbial extract library and the development of shark nanobodies.

**Supplementary Information:**

The online version contains supplementary material available at 10.1186/s41232-022-00207-9.

## Background

The immune system consists of innate immunity and adaptive immunity [1]. Innate immune cells, such as macrophages and dendritic cells, recognize pathogen-associated molecular patterns (PAMPs), including lipopolysaccharides, flagellins and double-stranded RNAs, as well as damage-associated molecular patterns (DAMPs, also known as danger signals or alarmins) that initiate noninfectious inflammatory responses. They also exhibit cytotoxic activity against transformed cells and infected cells. This innate recognition is mediated by germ line–encoded, non-clonal immune receptors, such as pattern recognition receptors (PRRs) and natural killer receptors [2].

Adaptive immune cells undergo gene recombination and express a wide variety of antigen-specific receptors on their cell surface or secrete antibodies [3]. Defects in the adaptive immune system lead to a variety of autoimmune diseases, such as rheumatoid arthritis (RA), inflammatory bowel disease, multiple sclerosis, neuromyelitis optica, and psoriasis, in which the immune system, including T cells and antibodies, recognizes self rather than non-self antigens [4, 5]. In RA, for instance, the adaptive immune system recognizes self antigens and secretes mediators that target the synovium, causing joint inflammation and pain. To alleviate the symptoms, immunosuppressive chemicals and anti-inflammatory biologics are prescribed for RA patients.

In recent years, increasing attention has been paid to the role of the innate immune system, especially inflammasomes, in the development of autoimmune diseases. Since protein–protein interaction (PPI) plays an essential role in the assembly of inflammasomes in the cytoplasm, it is imperative to establish novel strategies to interfere with PPI in the innate immune cells in order to develop therapeutics against inflammatory diseases. In this review, we summarize the development of conventional small molecule drugs and biologics for autoimmune diseases and introduce the drug discovery system at Nagasaki University, focusing on marine-derived microbes and molecules as ideal sources for the development of novel therapeutics, including mid-size molecule drugs and nanobodies.

## Main text

### Non-steroidal anti-inflammatory drugs

RA is an autoimmune and inflammatory disease characterized by pain, swelling, and stiffness in the joints, as well as fever, fatigue, and weakness. Humans have suffered from RA for thousands of years, and rheumatic pain was one of the most distressing symptoms to be relieved. During early human history, decoctions or extracts of herbs or plants played an important role in the alleviation of pain and fever. It is conceivable that salicylates were contained in some decoctions or extracts of herbs, such as the bark of willow tree (*Salix*), bark of poplar (*Popuplus*), and flower of meadowsweet (*Spirea*). The Ebers papyrus is the first record of a therapy harnessing herbal decoctions and is approximately 3500 years old [6]—it indicated that plant extracts were used as anti-inflammatory analgesics and antipyretic in ancient civilizations. In ancient Greece (approximately 2400 years ago), Hippocrates recommended the use of the bark of willow tree for the relief of rheumatic pain and fever.

In 1763, the first clinical report was published on the treatment of pain and fever with willow bark remedies. A pharmacologically active ingredient was isolated from the bark of the willow tree and named “salicin” between 1826 and 1829—this laid the foundation for the scientific development of anti-inflammatory and antipyretic drugs (Supplementary Fig. 1A) [7]. Salicin is an alcoholic β-glucoside prodrug and can be hydrolyzed into glucose and salicylic alcohol. Salicylic alcohol is further metabolized to salicylic acid, a pharmacologically active form. Between 1835 and 1838, the pure form of salicylic acid was prepared from salicylaldehyde derived from meadowsweet and also salicin from willow bark.

Salicylic acid was chemically synthesized in 1852 and acetylsalicylic acid in 1853 [8]. The antipyretic effects of synthetic salicylic acid were reported in 1875 and the anti-rheumatic fever effect in 1876 [7]. Since salicylic acid is bitter and toxic, causing stomach irritation, nausea, vomiting, and hearing disorders, acetylsalicylic acid was superior to salicylic acid as a therapeutic for rheumatic diseases. Felix Hoffmann improved the synthetic pathway to acetylsalicylic acid and confirmed a reduced toxicity and palatable profile of the derivative in 1897 [9]. Acetylsalicylic acid was one of the most widely used drugs in the last century and is still on the market under the trademark of “Aspirin” (*A* for acetyl, *spir* for Spirsäure, and *in* as a popular suffix for drugs).

Acetylsalicylic acid is a prototype of non-steroidal anti-inflammatory drugs (NSAIDs) that have analgesic, antipyretic, and anti-inflammatory effects and have been used as remedies for the treatment of a variety of symptoms, such as autoimmune inflammatory responses. Even though the mechanism by which acetylsalicylic acid alleviates rheumatic pain and fever had not been elucidated, a variety of NSAIDs were synthesized between 1961 and 1969, including diclofenac, flurbiprofen, ibuprofen, indometacin, ketoprofen, naproxen, piroxicam, and sulindac (Supplementary Fig. 1B) [5, 10]. Although the chemical structures of NSAIDs are diverse, they share the same therapeutic properties as well as side effects, especially gastric irritation.

In 1971, Vane and his colleagues demonstrated that acetylsalicylic acid as well as other NSAIDs inhibited an enzyme involved in the synthesis of prostaglandins, now called cyclooxygenase-1 (COX-1). The presence of another prostaglandin synthase was first suggested in 1972 [11, 12] and a gene encoding cyclooxygenase-2 (COX-2) was cloned in the early 1990s [13–18]. These findings clearly showed that the therapeutic effects of NSAIDs are ascribable to the inhibition of prostaglandin synthesis.

Whereas COX-1 is constitutively expressed in most tissues and is involved in the synthesis of prostaglandins and thromboxane A2 in physiological processes, the expression of COX-2 is induced by inflammatory mediators [10]. Since conventional NSAIDs inhibit both COX-1 and COX-2, many physiological processes are inhibited, resulting in adverse reactions such as gastrointestinal toxicities. Based on this finding, selective inhibitors of COX-2 were screened, designed, and synthesized, leading to the development of therapeutics specific for tissues undergoing inflammation. Representative COX-2 selective inhibitors include celecoxib, etoricoxib, lumaricoxib, parecoxib, rofecoxib, and valdecoxib as shown in Supplementary Fig. 2 [10].

### Glucocorticoids

As NSAIDs were developed empirically, glucocorticoids were also developed on the basis of empirical evidence showing that pregnancy and jaundice had beneficial effects on RA. During the study to elucidate the relationship between jaundice/pregnancy and RA, it was also found that temporary remissions of RA were frequently induced by anesthesia and surgical operation that might stimulate the adrenal cortices. Based on these clinical findings, it was conjectured that the adrenal cortices secreted a factor that might alleviate the symptoms of rheumatic diseases. Finally, 17-hydroxy-11-dehydrocorticosterone (a type of glucocorticoid, also known as cortisone) was found to be effective for patients with RA [19]. Cortisol (also known as hydrocortisone) is released from the adrenal cortex in the adrenal gland; cortisone is a prodrug of cortisol.

Glucocorticoids are a class of steroid hormones that bind to the glucocorticoid receptor and regulate glucose metabolism. In addition, glucocorticoids inhibit the proliferation of immune effector cells via the inhibition of cell signaling pathways such as AP-1 and NF-κB and negatively regulate immune responses. Since it was difficult to prepare natural glucocorticoids, much effort was devoted to the synthesis of glucocorticoid derivatives between the 1950s and 1980s. These synthetic glucocorticoids include betamethasone, cortisone, dexamethasone, methylprednisolone, and prednisone (Supplementary Fig. 3). Prednisone is a prodrug and can be converted by the enzyme in the liver to prednisolone, a pharmacologically active form. Although glucocorticoids are highly effective in the treatment of rheumatic diseases, they often induce adverse reactions, such as infection, gastrointestinal irritation, and bone damage, and the prolonged usage of glucocorticoids is not recommended in the treatment of RA.

### Disease-modifying anti-rheumatic drugs

The principal mechanism underlying the usage of glucocorticoids in the treatment of rheumatic diseases is suppression of the immune system. Since it was demonstrated that methotrexate could suppress the immune system, the effect of the compound on psoriatic arthritis and RA was examined in the 1960s. As expected, methotrexate improved the symptoms of the diseases. In addition, the therapeutic could be used for a prolonged period, unlike glucocorticoids [20].

Methotrexate is a prototype of disease-modifying anti-rheumatic drugs (DMARDs) and inhibits dihydrofolate reductase, which is essential for the synthesis of DNA and RNA, resulting in the suppression of immune effector cells and pro-inflammatory cytokines. The efficacy of methotrexate in the treatment of RA is, however, not satisfactory, leading to the synthesis of a variety of DMARDs, such as leflunomide, hydroxychloroquine, cyclophosphamide, sulfasalazine, and cyclosporine, as shown in Supplementary Fig. 4.

Since inhibitors of the enzymes involved in DNA and RNA synthesis negatively regulate the proliferation and function of fast-dividing cells such as lymphocytes, leading to immune suppression, a variety of inhibitors were examined to determine if they could be used as DMARDs in the treatment of rheumatic diseases. Leflunomide is an isoxazole derivative that inhibits the dihydroorotate dehydrogenase involved in the conversion of dihydroorotate to orotate coupled with the electron transfer from quinone to quinol, which is essential for both pyrimidine synthesis and the electron transfer chain. The compound is a prodrug and metabolized into teriflunomide, which is responsible for the inhibition of NF-κB signaling required for pro-inflammatory cytokines, including tumor necrosis factor-α (TNF-α) as well as the production of local metalloproteinases causing joint destruction [21].

Hydroxychloroquine is a 4-aminoquinoline derivative that exhibits anti-malarial activity and modulates immune responses. This drug is also clinically effective in the treatment of patients with RA, although the precise mechanism for the anti-rheumatic activity has not been elucidated yet. It is, however, evident that hydroxychloroquine suppresses activated immune effector cells and reduces the production of pro-inflammatory cytokines [22].

Cyclophosphamide is an alkylating agent used for the treatment of malignancy. Since the drug suppresses immune responses, it can be used to treat patients with autoimmune diseases. Because of its possible toxicity, cyclophosphamide is used for severe autoimmune diseases when conventional DMARDs are ineffective.

Sulfasalazine is metabolized into sulfapyridine and 5-aminosalicylic acid, which are absorbed in the intestine. Although the precise mechanism of the drug has not yet been deciphered, one possible mechanism is the inhibition of prostaglandin synthesis, leading to the anti-inflammatory effect. It was also suggested that the drug inhibits folate-dependent enzymes, resulting in the suppression of immune effector cells [23].

Cyclosporine is an inhibitor of calcineurin and impairs the function of effector T cells, leading to the inhibition of pro-inflammatory cytokine production [24].

### Biologics for autoimmune diseases

Although combinations of conventional small molecule drugs, such as NSAIDs, glucocorticoids, and DMARDs, are effective in the alleviation of symptoms of autoimmune diseases such as RA and psoriasis; the diseases are progressive and are generally associated with a reduced quality of life and loss of work capacity that places a substantial burden on patients as well as healthcare systems. An improved understanding of the pathogenesis of autoimmune diseases and advances in molecular biology techniques, however, led to the development of biologics such as chimera proteins and monoclonal antibodies.

**Fig. 1:**
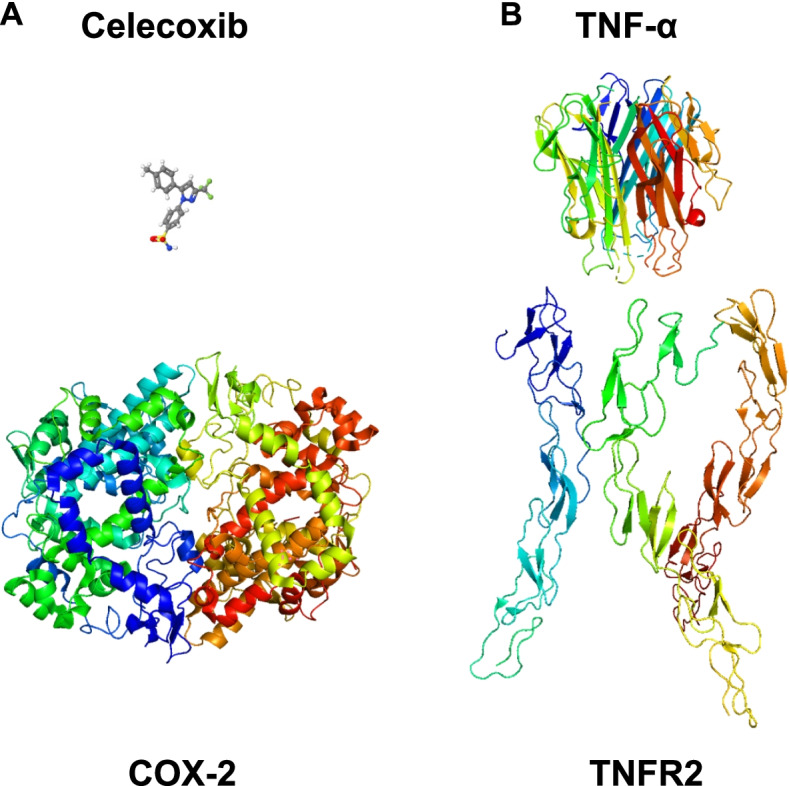
Comparison between the interface of a conventional small molecule drug and its target enzyme and that of a biologic and its receptor. **A** Interaction between celecoxib, a conventional small molecule drug, and COX-2. Since the molecular weights of most conventional drugs are less than 500 Dalton, the interfaces between the small molecule drugs and their target proteins are relatively small. **B** Interaction between TNF-α and the extracellular domain of TNFR2. Since TNF-α is a proteinaceous ligand and the interface between TNF-α and one of its receptor TNFR2 is large, the inhibitors should be large enough to interfere with the PPI

Since TNF-α plays an essential role in the onset of inflammatory symptoms in autoimmune diseases, anti-TNF-α therapies targeting the interaction between TNF-α and TNF-α receptors 1 (TNFR1) and 2 (TNFR2) are attractive measures to treat patients [25]. When it comes to the interaction between conventional small molecule drugs and their receptors, it is relatively easy to interfere with the ligand binding by small molecule inhibitors, since areas of interaction between the ligands and their receptors are relatively small [26]. In contrast, the TNF-α/TNFR complex has a significantly larger buried surface area than the conventional drug/receptor complex such as the celecoxib/COX-2 complex (Fig. 1) [27–33]. It is thus necessary to develop bulky inhibitors such as chimera receptor proteins and monoclonal antibodies for inhibition of the TNF-α/TNFR complex formation.

The first clinically approved anti-TNF-α biologic was a chimera or fusion protein consisting of the extracellular domain of TNFR and a constant domain of human immunoglobulin G1 (IgG1). Since the extracellular domain of TNFR binds to TNF-α, the fusion protein can functionally neutralize TNF-α. Subsequently, monoclonal antibodies specific to TNF-α were approved by the United States Food and Drug Administration (FDA). In the development of infliximab, mouse anti-human TNF-α monoclonal antibody was first established and the constant domains of the mouse antibody were replaced with the corresponding constant domains of human antibody. Adalimumab is a human monoclonal antibody that specifically binds to TNF-α [34].

In addition to TNF-α, other factors are also involved in the pathogenesis of autoimmune diseases. The major targets of biologics are interleukin-1β (IL-1β)/IL-1 receptor (IL-1R), IL-6/IL-6 receptor (IL-6R), and antigen-producing B cells (plasma cells). Anakinra is a modified human IL-1 receptor antagonist protein for the treatment of RA. Canakinumab is a human monoclonal antibody specific for IL-1β and is used for autoinflammatory diseases such as cryopyrin-associated periodic syndrome and Still’s disease. Rilonacept is a chimera protein consisting of the IL-1β-binding domains of the IL-1R subunits (IL-1R1 and IL-1R accessory protein) and the fragment-crystallizable domain (Fc) of human IgG1, which specifically binds IL-1 [35].

Since IL-6 is a mediator of fever and acute-phase responses, the blockade of IL-6/IL-6R alleviates the symptoms in autoimmune diseases. Tocilizumab is a humanized anti-IL-6R monoclonal antibody and is used for the treatment of RA in combination with methotrexate. When RA patients are unresponsive to anti-TNF-α therapies and DMARDs, B cell depletion is sometimes beneficial. Rituxan is a chimeric monoclonal antibody specific for cluster of differentiation 20 (CD20) expressed on B cells and can efficiently eliminate B cells through the induction of apoptosis mediated by antibody-dependent cellular cytotoxicity and complement-dependent cytotoxicity [35].

### Inflammasomes

The innate immune system is involved in the first line of defense against infections and sterile insults through the recognition of PAMPs and DAMPs by PRRs [36]. PRR sensing leads to the formation of protein complexes called inflammasomes, including nucleotide-binding domain, leucine-rich repeat-containing receptor (NLR) or nucleotide-binding oligomerization domain-like (NOD-like) receptor (NLR) family pyrin domain containing 1 (NLRP1), NLRP3, NLR family caspase activation, and recruitment domain (CARD)-containing protein 4 (NLRC4), absent in melanoma 2 (AIM2) and pyrin. The inflammasomes are composed of a sensor, apoptosis-associated speck-like protein containing a CARD (ASC), and caspase-1. When the inflammasomes are assembled, caspase-1 is activated, resulting in the conversion of pro-IL-1β and pro-IL-18 to mature IL-1β and IL-18. In addition, the activated caspase-1 cleaves gasdermin D and the N-terminal domain of gasdermin D forms oligomeric pores in the membrane, which allows the release of mature IL-1β and IL-18 [37].

Since the inflammasomes are sensors that normally recognize infections and sterile insults, the release of IL-1β and IL-18 contributes to the first line of defense and is beneficial to our health [38]. However, gain-of-function mutation in the inflammasomes can lead to autoinflammatory diseases. Pyrin is encoded by the *MEFV* gene and a sensor of bacterial infections, such as *Yersinia pestis*. Gain-of-function mutation in the *MEFV* gene can lead to familial Mediterranean fever (FMF). This monogenic autoinflammatory disease is characterized by recurrent episodes of fever and overproduction of IL-1β. Since the mutation in pyrin results in the formation of the pyrin inflammasome complex, it is necessary to disassemble the multi-protein complex in the treatment of FMF [39].

In anti-TNF-α therapies, TNF-α is released extracellularly. Intravenously administered anti-TNF-α biologics can, therefore, encounter TNF-α molecules and neutralize their effector functions. In contrast, pyrin inflammasomes reside in the cytoplasm and intravenously injected bulky biologics such as anti-pyrin or anti-ASC monoclonal antibodies do not permeate the cell membranes and disassemble the multi-protein complexes. On the contrary, conventional synthetic compounds can be internalized into the cells, whereas it is generally difficult for the small molecules to interfere with PPI. It is thus imperative to develop mid-size molecules or molecularly engineered nanobodies as next-generation therapeutics for the treatment of autoinflammatory diseases such as FMF and Still’s disease.

### Marine microbes as sources of therapeutics for autoimmune diseases

At Nagasaki University, we have been collecting marine microbes and preparing an original marine microbial extract library in an attempt to discover mid-size therapeutics as well as conventional small-size molecules. Nagasaki Prefecture is located in the west of Japan and is surrounded by seas, bays, and inlets, giving it the second longest coastline of all of the Japanese prefectures. It is blessed with natural resources, such as marine animals, seaweeds, and marine microbes.

Life on Earth may have started in the oceans around 4 billion years ago [40]. At present, oceans cover almost three-quarters of the Earth’s surface and represent 99% of the living space by volume, leading to great biodiversity. The number of marine microbes exceeds 3.6 × 10^29^ cells, with a total cellular carbon content of 3 × 10^17^ g, and most of life’s genetic and metabolic diversity is derived from microbes [41, 42]. As a result, many natural products can elicit a wide spectrum of biological activities by interacting with a variety of biomolecules, such as enzymes and receptors [43].

Natural products have been a major source of therapeutic leads over the last 40 years. According to a comprehensive review of FDA-approved drugs filed between January 1981 and September 2019, 3.8% of all drugs are unaltered natural products, 0.8% are botanical drugs, 18.9% are natural product derivatives, 3.2% are synthetic drugs with a natural product pharmacophore, and 22.5% are natural product mimetics, demonstrating that 49.2% of all drugs are inexorably linked to natural products [44]. Historically, the majority of medicines were decoctions or extracts of herbs and plants that were accessible to humans. Due to the development of culture systems for marine microbes and identification methods, it is now possible to systematically collect marine microbes and prepare marine microbial extract libraries.

In the development of therapeutics, one of the most important things is to find novel backbone structures of compounds or biomolecules. It is thus essential to use original drug libraries with diverse chemical space and molecular weights [45, 46]. Since marine microbes contain a wide variety of biomolecules, including mid-size molecules as well as relatively small compounds, it is a practical and ideal strategy to develop a system using an original marine microbial extract library for the discovery of novel pharmaceutical leads and therapeutic molecules [47, 48].

**Fig. 2:**
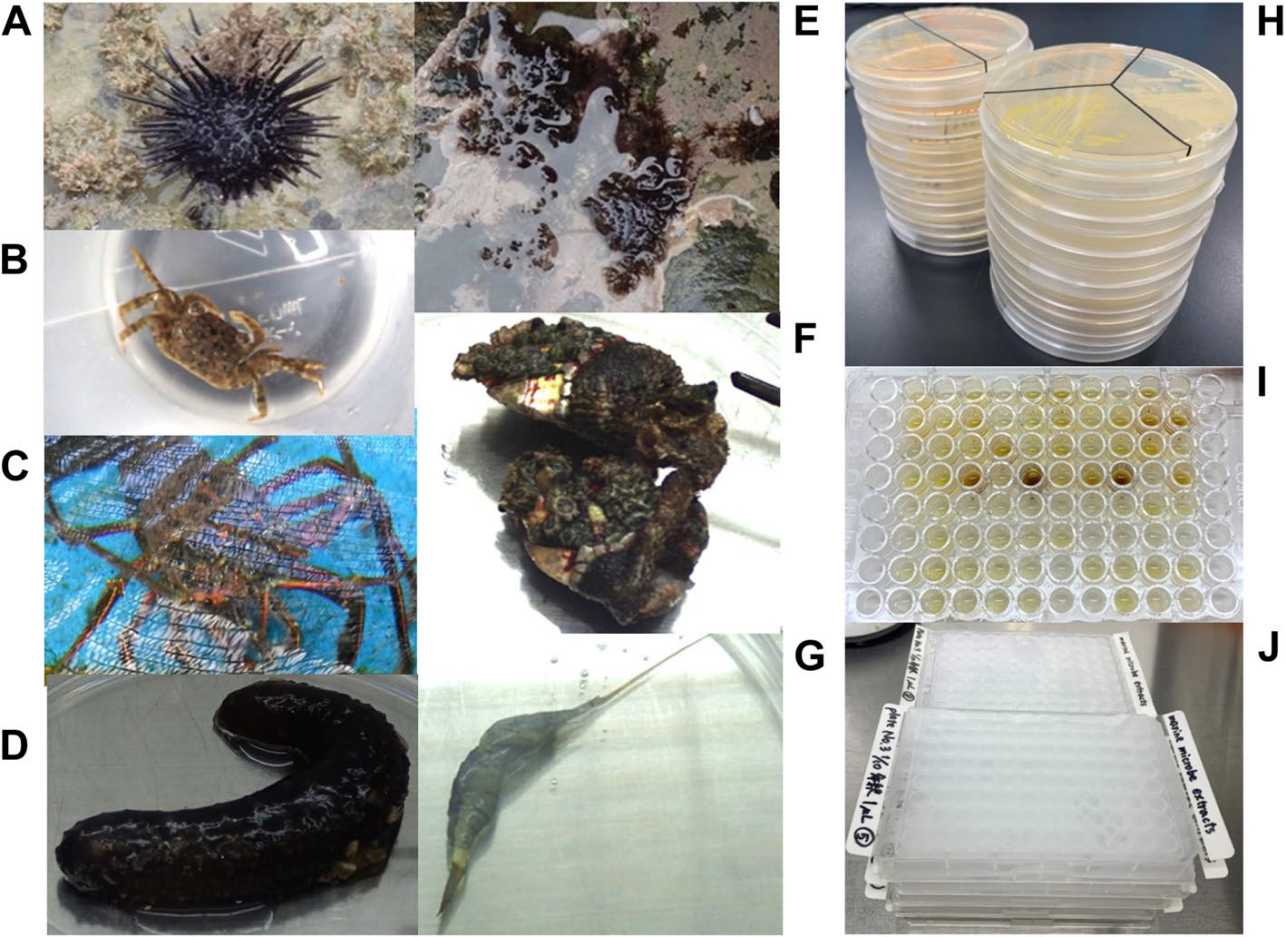
Preparation of the marine microbial extract library. Collection of marine animals from the coasts of Nagasaki Prefecture: **A** sea urchin, **B** crab, **C** lobster, **D** sea cucumber, **E** sea anemone, **F** oyster, and **G** shrimp. **H** Isolation of marine microbes from various marine animals. Samples from a variety of marine animals are streaked out onto marine agar plates or seawater plates and marine microbes are isolated. **I** Preparation of marine microbial extracts. The isolated marine microbes are grown in marine broth or seawater broth and the marine microbial extracts are prepared as shown in Fig. 3. **J** Development of a marine microbial extract library. Marine microbial extracts are dissolved in DMSO at a concentration of 10 or 100 mg/ml and dispensed into 96-well plates

In order to efficiently collect marine microbes, we set out to isolate marine microbes along the coasts of Nagasaki Prefecture. Although we initially attempted to isolate marine microbes from seawater, the efficiency of microbial colony formation was too low. As such, we collected sea animals and isolated marine microbes mainly from the alimentary canal. As shown in Fig. 2A–G, many kinds of sea animals were collected from the coasts, such as sea urchin, crab, lobster, sea cucumber, sea anemone, oyster, and shrimp. The alimentary canal samples were streaked out onto marine agar and seawater agar plates, which were incubated at 26 °C for several days (Fig. 2H). After colonies were isolated based on colony morphology and pigmentation, the marine microbes were cultured in a large scale and their extracts were prepared. Finally, the extracts were dispensed into multi-well plates and stored at − 30 °C (Fig. 2I and J).

**Fig. 3:**
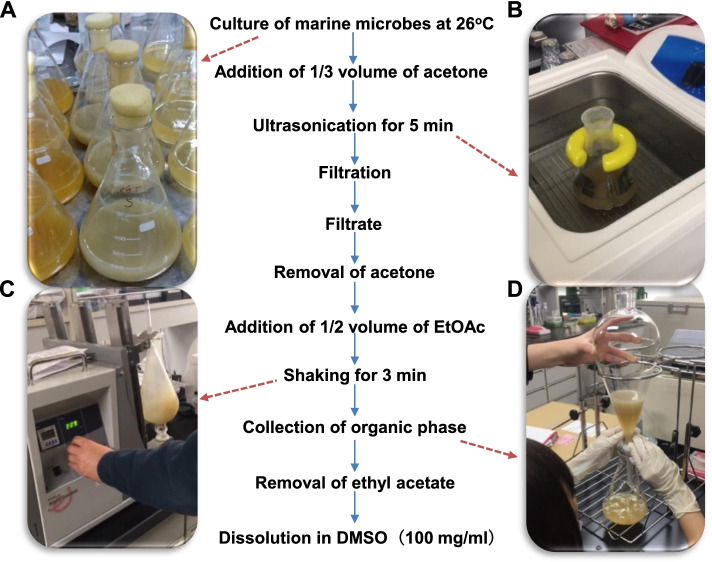
Preparation of marine microbial extracts. **A** Culture of marine microbes. Isolated marine microbes are grown in marine broth or seawater broth in Erlenmeyer flasks at 26 °C. **B** Sonication of marine microbial culture. Marine microbial culture is treated with acetone and sonicated in a water bath. **C** Extraction of sonicated marine microbial culture with EtOAc. After the acetone is evaporated, EtOAc is added to the marine microbial sonicate. The marine microbial sonicate/EtOAc suspension is transferred into a separatory funnel, which is vigorously shaken for 3 min. **D** Collection of EtOAc-extracted samples. After the water-soluble layer is drained, the organic phase is collected, evaporated, and dissolved in DMSO to give a final concentration of 10 or 100 mg/ml

A scheme for the preparation of marine microbial extracts is illustrated in Fig. 3. Briefly, marine microbes were grown in marine broth or seawater broth in 2 L Erlenmeyer flasks at 26 °C for several days (Fig. 3A). To the marine microbial culture was added 1/3 volume of acetone, which was sonicated for 5 min (Fig. 3B). The sonicated samples were filtered through a filter paper and the filtrate was subjected to vacuum evaporation to remove acetone. To the residue was added 1/2 volume of ethyl acetate, and the mixture was shaken in separatory funnels for 3 min (Fig. 3C). After the aqueous phase was drained (Fig. 3D), the organic phase was collected and subjected to vacuum evaporation to remove ethyl acetate. Dimethyl sulfoxide was added to the extract to a concentration of 100 mg/ml, which was dispensed into cryovials and stored at − 30 °C. The water phase was also concentrated and stored as aqueous extract samples.

The resulting marine microbe library should contain a wide variety of compounds with novel backbone structures as well as mid-size biomolecules, such as derivatives of peptides, carbohydrates, lipids, and nucleic acids. Over the last 100 years, many laboratories have screened extracts of microbes in terrestrial environments for the development of therapeutics for inflammatory diseases, including autoimmune diseases, autoinflammatory diseases, fever, and pain. In contrast, marine microbial extracts have not been extensively explored as a source of drug discovery, because access to marine microbe libraries has been difficult due to their scarcity. At Nagasaki University, we have been developing a marine microbial extract library-based platform for academic drug discovery and are providing our original library for academic use. We hope that our original marine microbial extract library will be a useful tool for drug discovery in academia and contribute to the development of therapeutics for various autoimmune diseases.

### Nanobodies derived from heavy-chain antibodies

The targets of conventional small molecule drugs are proteins that interact with relatively small molecules, such as membrane-bound receptors that recognize small molecule ligands, and intracellular enzymes that catalyze the reactions involving small metabolites. Since the areas of interaction between enzymes/receptors and substrates/ligands are relatively small, the inhibitors can be small, leading to the successful development of conventional small-molecule drugs. Since humans have only approximately 20,000 genes in our genomes, the targets of conventional drugs are limited. In fact, a paucity of conventional drug targets has been recognized by researchers in pharmaceutical companies over the last three decades and the development of monoclonal antibodies has been the major pipeline since the mid-1990s. The targets of the current monoclonal antibody therapeutics are membrane-bound receptors that recognize extracellular soluble ligands, or soluble ligands that are recognized by cellular receptors. Since monoclonal antibodies cannot be internalized into cells, their major targets reside outside of the cells.

For autoinflammatory diseases, the drug targets are the PPIs inside the cells, such as the interaction between pyrin and ASC. Although hydrophobic small molecules can be readily internalized into the cytoplasm, the PPI is not efficiently inhibited by such conventional molecules, because areas of PPI are too large for the small molecules to interfere with the interaction. In contrast, human or humanized monoclonal antibodies are too large to be internalized into target cells and, therefore, fail to interfere with the intracellular PPI, even though the monoclonal antibodies can efficiently interfere with PPI outside of the cells.

As described in the previous section, mid-size marine biomolecules are possible candidates, since they might be able to permeate cell membranes and interfere with the intracellular PPI. In addition, nanobodies, also known as single-domain antibodies, are also promising candidates as inhibitors of intracellular PPI, because modified nanobodies can be internalized into cells. The molecular weights of nanobodies are typically 12 to 15 kDa and are larger than mid-size marine biomolecules with a molecular weight of 1 to 3 kDa, suggesting that PPI inhibition by nanobodies is more efficient than that by mid-size biomolecules, once the inhibitors are internalized into cells.

Nanobodies are derived from the heavy-chain antibodies of camelids or the immunoglobulin new antigen receptors (IgNAR) of cartilaginous fish. The first heavy-chain antibodies were reported in 1993 [49]. In the late 1980s, there was a practical course aimed at purifying antibodies from human blood sera at the Free University of Brussels in Belgium. Since HIV infection was a growing concern in the world at that time, students preferred to analyze non-human sera. Incidentally, a sample of dromedary sera was found in a freezer and examined for the existence of antibodies. In addition to antibodies of known migration patterns on electrophoreses, they found smaller bands of antibody-like proteins. It was later found that the dromedary sera contained not only conventional antibodies, but also antibodies consisting of only two identical heavy chains, now called heavy-chain antibodies or heavy chain-only antibodies that lack two light chains found in conventional antibodies. Heavy-chain antibodies were also found in other species of camelids, such as Bactrian camels, alpacas, llamas, guanacos, and vicuñas. They are composed of two identical heavy chains, each consisting of a variable domain and two constant domains [50–52].

The diversity of heavy-chain antibodies is generated through recombination-activating gene (RAG)-mediated V(D)J recombination with terminal deoxynucleotidyl transferase (TdT)-mediated junctional diversification. They are also modified by activation-induced cytidine deaminase-catalyzed somatic hypermutation (SHM) [53]. The antigen recognition site resides in the single variable domain, which is called the variable domain of the heavy chain of the heavy-chain antibody (V_H_H). V_H_H is composed of a single domain and the molecular size is small enough to be expressed in *Escherichia coli*. Gene-engineered V_H_H is currently often called a nanobody.

In conventional antibodies, the antigen recognition module is composed of two non-covalently associated variable domains; one is a variable domain of the heavy chain (V_H_) and the other is that of the light chain (V_L_) [54]. Since the orientation of the two domains defines the antigen specificity in the V_H_/V_L_ complex, the two domains must be tightly interacted, which requires large hydrophobic surface areas in the two domains. It is of note that hydrophobic surface areas endow proteins with structural instability, making the V_H_/V_L_ complex unstable. In contrast, V_H_H, or nanobody, is a single domain protein with no large hydrophobic surface areas, leading to the structurally robust nature of nanobodies. In addition, the absence of hydrophobic regions contributes to the improved solubility and heat stability of nanobodies. Another advantage of nanobodies is their small size, which enables them to penetrate tissues and cells [55–57]. The addition of cell-penetrating peptides or hydrophobic chains may further increase cell permeability. It may thus be possible to develop therapeutic nanobodies that penetrate cells and interfere with the PPI. For example, cell-penetrating nanobodies that can interfere with the interaction between pyrin and ASC may be used for the treatment of patients with FMF.

### Shark nanobodies as therapeutics for autoimmune diseases

After the discovery of heavy-chain antibodies in camelids in 1993, other species of animals bearing an adaptive immune system were examined as to whether or not they have heavy-chain antibodies. In 1995, another heavy-chain antibody, IgNAR, was discovered in cartilaginous fish, such as sharks, rays, and skates. IgNAR consists of two identical heavy chains, each consisting of a variable domain (V_NAR_) and five constant domains (C1_NAR_, C2_NAR_, C3_NAR_, C4_NAR_, and C5_NAR_) [58–60]. V_NAR_ is involved in antigen binding, recognition, and specificity. In fact, the variable domain is formed through the somatic rearrangement of one variable segment, three diversity segments, and one joining segment. The joining ends are trimmed and further modified with concurrent N- and P-additions as well as antigen-driven SHM.

Since the most ancient adaptive immune system is found in cartilaginous fish, including sharks [61, 62], the evolutionary distance between human and shark immune system-related genes is much greater than that between human and camelid immune system-related genes, suggesting that high-affinity nanobodies could be obtained by immunizing sharks with human-derived antigens, when compared to immunizing camelids with the same antigens. The evolutionary distance is generally considered one of the most important factors for defining the antigenicity. In addition, the shark V_NAR_ complementarity-determining region 3 (CDR3) that is involved in antigen recognition is highly variable in size, indicating that the lengths of shark V_NAR_ CDR3 can be significantly longer than those of mammalian V_H_/V_L_ CDR3. Because the long CDR3 loops may access recessed epitopes that are not recognized by the canonical V_H_/V_L_ CDR3 loops, it might be possible to develop shark nanobodies that recognize protein targets that are not typically recognized by conventional mammalian monoclonal antibodies [63].

**Fig. 4:**
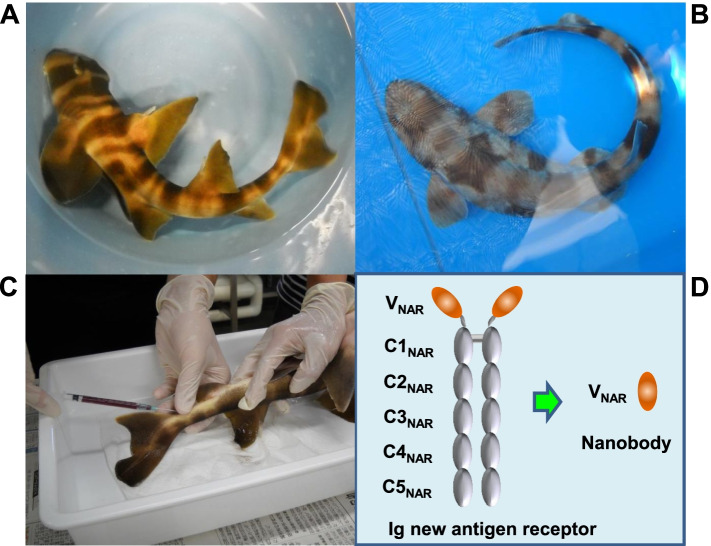
Preparation of shark nanobodies. **A** Japanese bullhead shark. *Nekozame* in Japanese; *Heterodontus japonicas*. **B** Cloudy catshark. *Torazame* in Japanese; *Scyliorhinus torazame*. **C** Blood collection from a Japanese bullhead shark. Blood is drawn through a vascular sinus behind the dorsal fin using a sterile disposable needle and syringe. **D** Schematic representation of shark Ig new antigen receptor (IgNAR) and shark nanobody. Shark IgNAR consists of two identical protein chains each with one variable domain (V_NAR_) and five constant domains (C1_NAR_–C5_NAR_)

Like camelid nanobodies, shark nanobodies are structurally stable, since a number of charged and hydrophilic amino acid side chains are located on the surface of the Ig scaffold and the CDR loops are fixed by intra-loop disulfide linkages and hydrogen bonds. Taken together, the structural findings show that shark-derived nanobodies can be promising candidates as therapeutic biomolecules for autoinflammatory diseases. In the Institute for East China Sea Research of Nagasaki University located on the coast line of Nagasaki City, we have established a shark farming system to stably provide Japanese bullhead sharks (*nekozame* in Japanese; *Heterodontus japonicas*) and cloudy catsharks (*torazame* in Japanese; *Scyliorhinus torazame*) to researchers who are interested in the development of shark nanobodies (Fig. 4A and B). Shark peripheral blood can be drawn through a vascular sinus behind the dorsal fin using a sterile disposable needle and syringe, from which V_NAR_ genes can be extracted (Fig. 4C). As shown in Fig. 4D, IgNARs derived from Japanese bullhead sharks and cloudy catsharks consist of two identical protein chains, each with V_NAR_ and C1_NAR_–C5_NAR_. After immunization of the sharks with antigens, the genes encoding the V_NAR_ domains can be isolated from peripheral blood and the V_NAR_ can be expressed in *E*. *coli*. We are currently trying to establish a system for the expression and refolding of *E*. *coli* inclusion bodies at the Center for Medical Innovation of Nagasaki University located in downtown Nagasaki City. Since it is necessary to immunize sharks several times over a period of several months to obtain high-affinity IgNARs, we are constructing a shark aquaculture system at Nagasaki University Hospital. We hope that our system for developing shark nanobodies will be used by as many researchers as possible and contribute to the development of novel nanobodies for the treatment of autoimmune diseases in the future.

## Conclusions

The development of NSAIDs, glucocorticoids, DMARDs, monoclonal antibodies, and their combination therapies has greatly improved the quality of life of a considerable number of patients with autoimmune diseases. The targets of the conventional small-molecule drugs and biologics are generally intracellular enzymes or receptors that recognize small-molecule substrates or ligands, or membrane-bound receptors or extracellular proteinaceous factors. In cases of autoinflammatory diseases such as FMF, however, the potential drug targets should be intracellular PPI, such as the interaction between pyrin and ASC. For a novel category of targets, a novel class of therapeutics is required. If this is the case, shark nanobodies have properties that make them candidates as inhibitors of intracellular PPI. It is our hope that shark nanobodies will provide therapeutic benefits to patients with autoinflammatory diseases.

## Supplementary Information

**Additional file 1: Supplementary Fig. 1.** Structure of non-steroidal anti-inflammatory drugs. (A) Structure of salicin-related compounds. Salicin is a water-soluble β-glucoside and is produced in willow bark. Salicin is metabolized into salicylic acid, an active form of the willow bark decoction or extract. Acetylsalicylic acid is known as Aspirin and is one of the most widely used medications for pain, fever, and inflammation. (B) Representative non-steroidal anti-inflammatory drugs. Non-steroidal anti-inflammatory drugs (NSAIDs) developed in the 1960s inhibit both cyclooxygenase-1 (COX-1) and COX-2 and decrease inflammation. **Supplementary Fig. 2.** Structure of COX-2 inhibitors. COX-2 selective inhibitors are non-steroidal anti-inflammatory drugs (NSAIDs) that decrease inflammation and have reduced risk of adverse gastrointestinal effects. **Supplementary Fig. 3.** Structure of glucocorticoids. Glucocorticoids are a class of corticosteroids that bind to the glucocorticoid receptor involved in the regulation of glucose metabolism. Glucocorticoids suppress the immune system and reduce inflammation. **Supplementary Fig. 4.** Structure of representative disease-modifying anti-rheumatic drugs. Disease-modifying anti-rheumatic drugs (DMARDs) comprise a group of compounds that alleviate rheumatoid arthritis by slowing down the progression of the disease.

## Data Availability

Not applicable.

## Abbreviations

AIM2: Absent in melanoma 2
ASC: Apoptosis-associated speck-like protein containing a CARD
CD20: Cluster of differentiation
CARD: Caspase activation and recruitment domain
C1_NAR_: Constant domain 1 of IgNAR
DAMPs: Damage-associated molecular patterns
DMARDs: Disease-modifying anti-rheumatic drugs
Fc: Fragment-crystallizable domain
FDA: Food and Drug Administration
FMF: Familial Mediterranean fever
V_H_H: Heavy-chain antibody or heavy chain-only antibody
IgG1: Immunoglobulin G1
IgNAR: Immunoglobulin new antigen receptor
IL-1: Interleukin-1
NLR: Nucleotide-binding domain, leucine-rich repeat-containing receptor, or NOD-like receptor
NLRC4: NLR family CARD-containing protein 4
NLRP1: NLR family pyrin domain containing 1
NOD: Nucleotide-binding oligomerization domain
NSAIDs: Non-steroidal anti-inflammatory drugs
PAMPs: Pathogen-associated molecular patterns
PRRs: Pattern recognition receptors
PPI: Protein—protein interaction
RA: Rheumatoid arthritis
RAG: Recombination-activating gene
SHM: Somatic hypermutation
TdT: Terminal deoxynucleotidyl transferase
TNF-α: Tumor necrosis factor-α
TNFR: Tumor necrosis factor-α receptor
V_H_: Variable domain of a heavy chain
V_L_: Variable domain of a light chain
V_NAR_: Variable domain of IgNAR
