# Supplementary material for: Marine-derived microbes and molecules for drug discovery
Source: Inflamm Regen. 2022 Jun 3;42:18. doi: 10.1186/s41232-022-00207-9 (PMC9164490; doi:10.1186/s41232-022-00207-9)
Supplement: Supplementary file 1 — Additional file 1: Supplementary Fig. 1. Structure of non-steroidal anti-inflammatory drugs. (A) Structure of salicin-related compounds. Salicin is a water-soluble β-glucoside and is produced in willow bark. Salicin is metabolized into salicylic acid, an active form of the willow bark decoction or extract. Acetylsalicylic acid is known as Aspirin and is one of the most widely used medications for pain, fever, and inflammation. (B) Representative non-steroidal anti-inflammatory drugs. Non-steroidal anti-inflammatory drugs (NSAIDs) developed in the 1960s inhibit both cyclooxygenase-1 (COX-1) and COX-2 and decrease inflammation. Supplementary Fig. 2. Structure of COX-2 inhibitors. COX-2 selective inhibitors are non-steroidal anti-inflammatory drugs (NSAIDs) that decrease inflammation and have reduced risk of adverse gastrointestinal effects. Supplementary Fig. 3. Structure of glucocorticoids. Glucocorticoids are a class of corticosteroids that bind to the glucocorticoid receptor involved in the regulation of glucose metabolism. Glucocorticoids suppress the immune system and reduce inflammation. Supplementary Fig. 4. Structure of representative disease-modifying anti-rheumatic drugs. Disease-modifying anti-rheumatic drugs (DMARDs) comprise a group of compounds that alleviate rheumatoid arthritis by slowing down the progression of the disease. [file 41232_2022_207_MOESM1_ESM.ppt]

## Slide 1
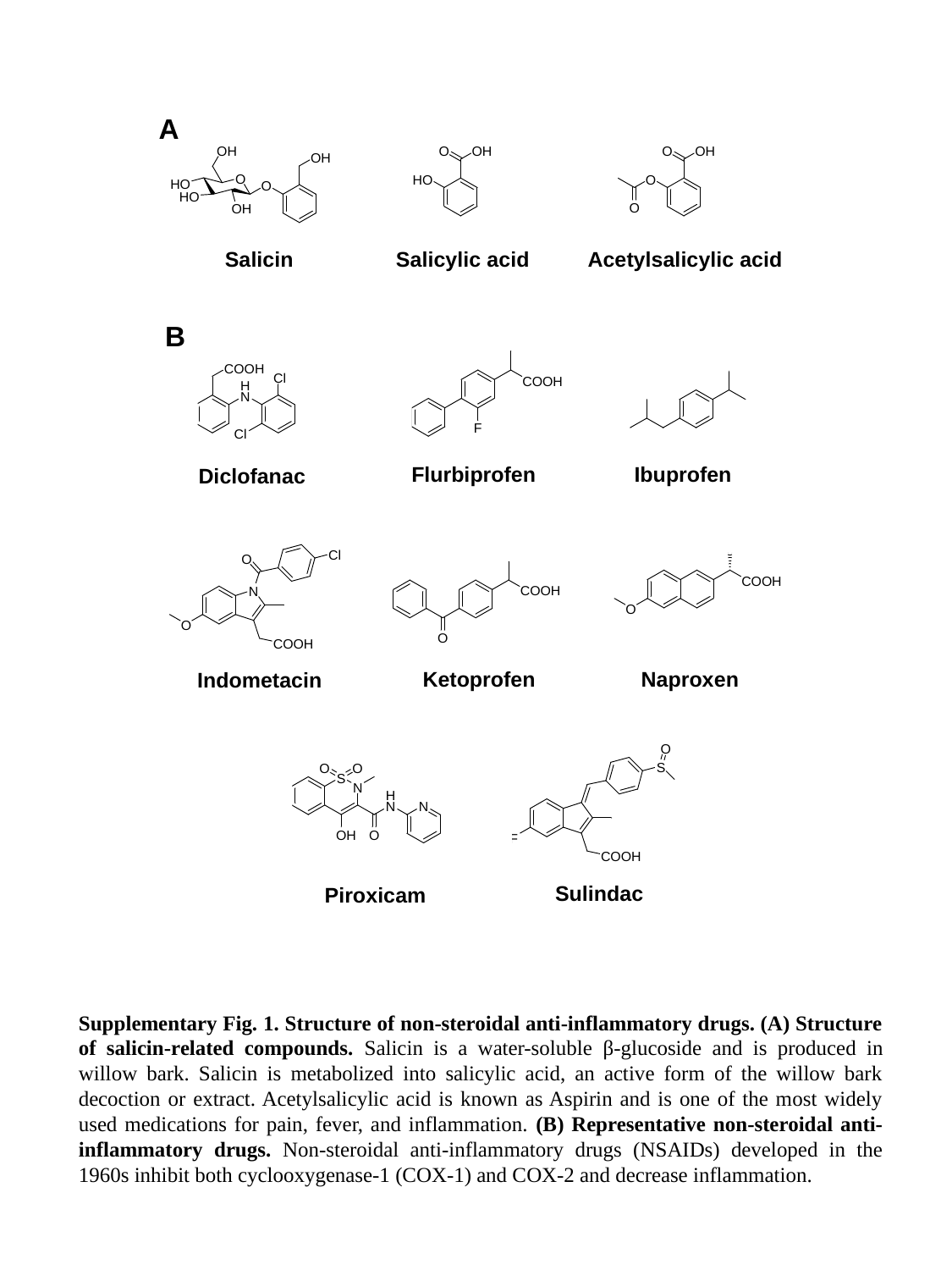

A
Salicin
Salicylic acid
Acetylsalicylic acid
B
Flurbiprofen
Ibuprofen
Diclofanac
Ketoprofen
Naproxen
Indometacin
Sulindac
Piroxicam
Supplementary Fig. 1. Structure of non-steroidal anti-inflammatory drugs. (A) Structure of salicin-related compounds. Salicin is a water-soluble β-glucoside and is produced in willow bark. Salicin is metabolized into salicylic acid, an active form of the willow bark decoction or extract. Acetylsalicylic acid is known as Aspirin and is one of the most widely used medications for pain, fever, and inflammation. (B) Representative non-steroidal anti-inflammatory drugs. Non-steroidal anti-inflammatory drugs (NSAIDs) developed in the 1960s inhibit both cyclooxygenase-1 (COX-1) and COX-2 and decrease inflammation.

## Slide 2
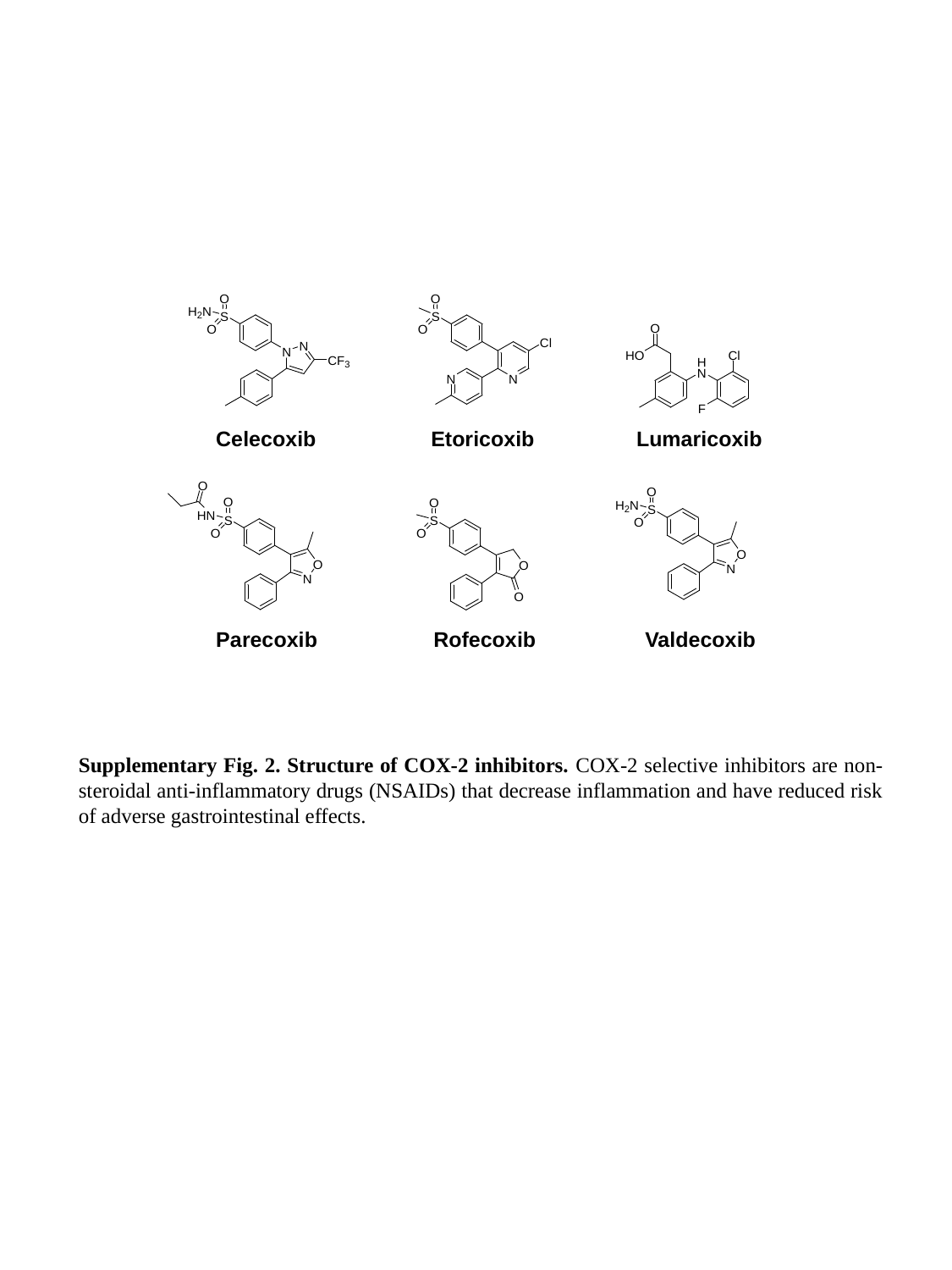

Celecoxib
Etoricoxib
Lumaricoxib
Parecoxib
Rofecoxib
Valdecoxib
Supplementary Fig. 2. Structure of COX-2 inhibitors. COX-2 selective inhibitors are non-steroidal anti-inflammatory drugs (NSAIDs) that decrease inflammation and have reduced risk of adverse gastrointestinal effects.

## Slide 3
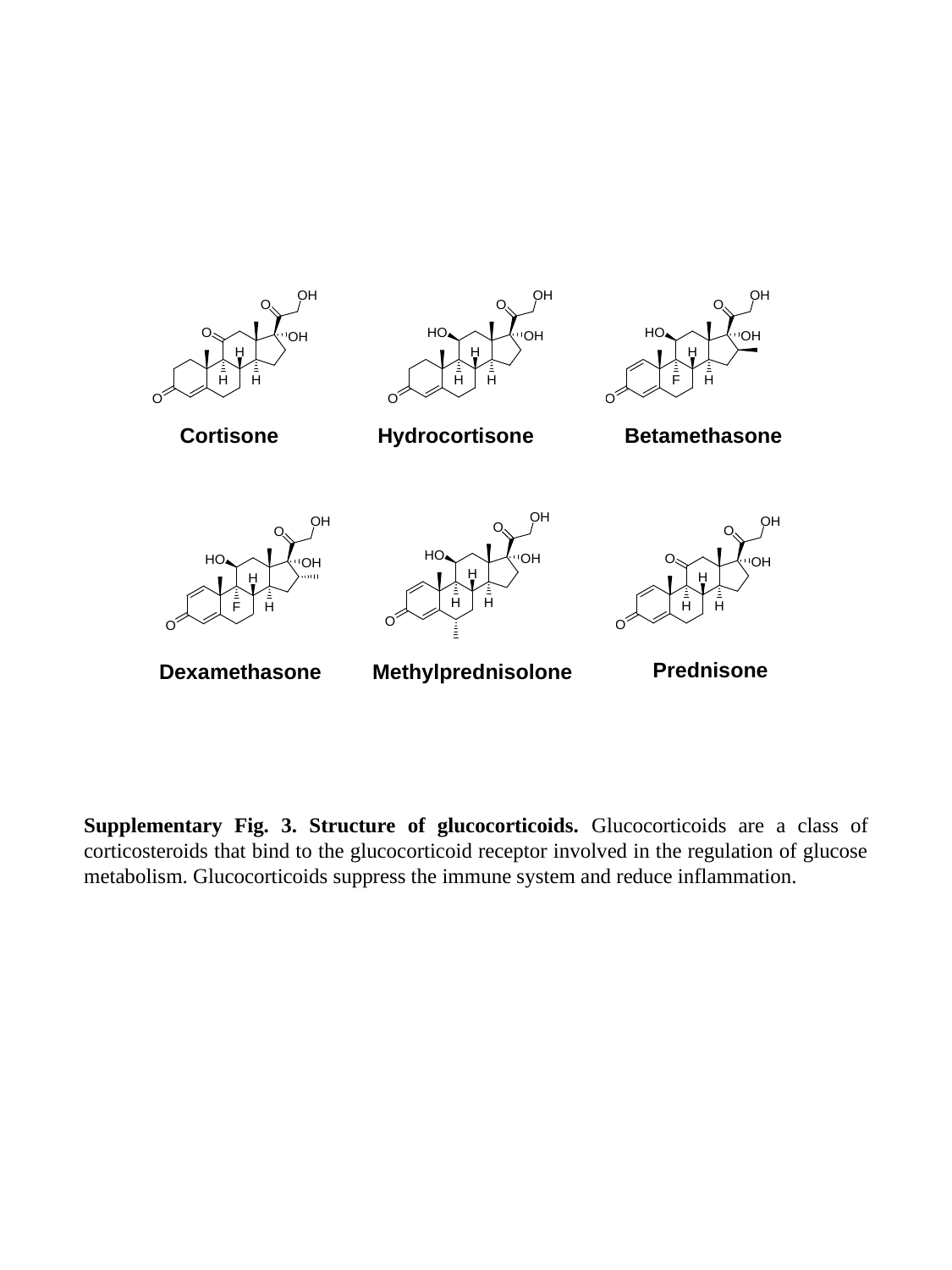

Cortisone
Hydrocortisone
Betamethasone
Prednisone
Dexamethasone
Methylprednisolone
Supplementary Fig. 3. Structure of glucocorticoids. Glucocorticoids are a class of corticosteroids that bind to the glucocorticoid receptor involved in the regulation of glucose metabolism. Glucocorticoids suppress the immune system and reduce inflammation.

## Slide 4
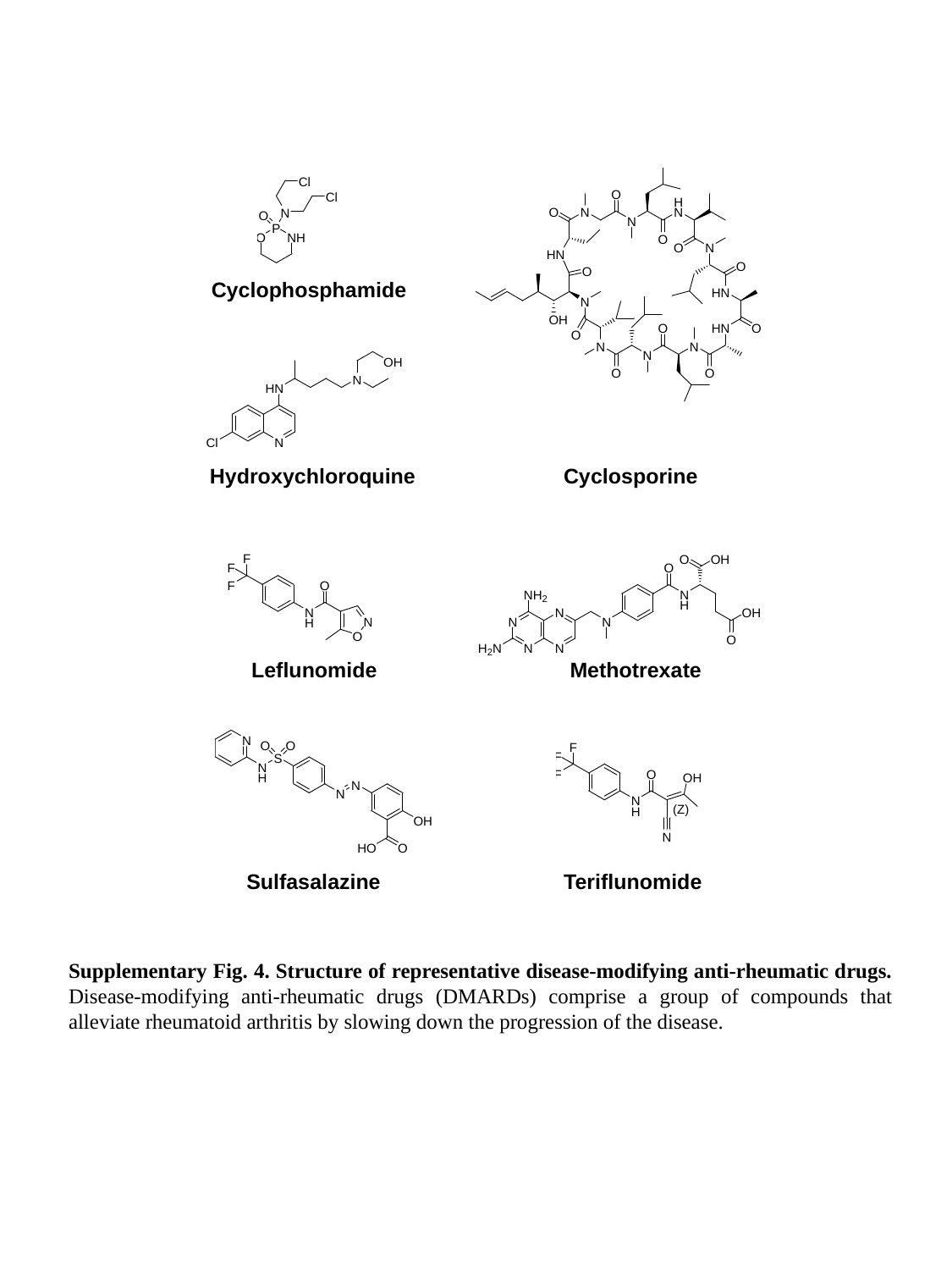

Cyclophosphamide
Hydroxychloroquine
Cyclosporine
Leflunomide
Methotrexate
Sulfasalazine
Teriflunomide
Supplementary Fig. 4. Structure of representative disease-modifying anti-rheumatic drugs. Disease-modifying anti-rheumatic drugs (DMARDs) comprise a group of compounds that alleviate rheumatoid arthritis by slowing down the progression of the disease.
